# Mathematical Models of Grinding Forces in the Hob Cutter Sharpening Process

**DOI:** 10.3390/ma18010138

**Published:** 2025-01-01

**Authors:** Błażej Witkowski, Wojciech Stachurski, Witold Pawłowski, Małgorzata Sikora, Norbert Kępczak

**Affiliations:** Institute of Machine Tools and Production Engineering, Faculty of Mechanical Engineering, Lodz University of Technology, Stefanowskiego 1/15, 90-537 Lodz, Poland; blazej.witkowski@p.lodz.pl (B.W.); wojciech.stachurski@p.lodz.pl (W.S.); witold.pawlowski@p.lodz.pl (W.P.); malgorzata.sikora@p.lodz.pl (M.S.)

**Keywords:** grinding, hob cutter, sharpening of cutting tools, grinding force, modelling of grinding force

## Abstract

The article presents the results of research aimed at developing mathematical models for determining the components of grinding force occurring during the sharpening of the rake face of hob cutters. The development of the models was based on the results obtained during experimental tests conducted in the first stage of the research. The studies were carried out using a tool grinder and an aluminum oxide grinding wheel. During the tests, two components of the grinding force were measured, using a piezoelectric dynamometer. A sample made of HS6-5-2 high-speed steel was ground. Grinding was carried out using different sets of cutting parameters. Based on the obtained measurement results, two mathematical models were developed in the form of (1) a multiple regression function and (2) a polynomial function, enabling the calculation of the normal and the tangential force. The experimentally obtained results were then compared with those calculated based on the developed models, and the results of this comparison showed that the developed models provide a good basis for analyzing the sharpening process of hob cutters in terms of variable grinding parameters.

## 1. Introduction

In the era of mass industrial production of gears [1], the proper regeneration of hob cutters, cutting tools commonly used for manufacturing toothed elements, is of great importance [2]. This is because improperly selected grinding conditions during the sharpening of the rake surfaces of the cutters can adversely affect the technological properties of the surface layer, causing, among other things, high thermal stresses leading to a network of cracks [3,4], and structural changes significantly reducing the cutting properties of the edges [5] and deteriorating their operational properties [6].

Therefore, it is extremely important to consciously shape the surface layer during grinding. The means to achieve this is to assess the course of the machining process, and one of the most important elements allowing for such an assessment is the knowledge of cutting forces. The significance of such an assessment lies in the dependence of the adjustable machining values, such as grinding parameters, the geometry of the contact between the grinding wheel and the workpiece, the characteristics of the grinding wheel, and the material properties on the values of the occurring forces. Moreover, knowledge of the obtained values of the grinding force components allows, among other things, for the determination of the grinding force coefficient *μ*, which provides information about the grinding efficiency [7], and the calculation of the total grinding power *P*, enabling the determination of the grinding index *K_s_* [8]. Generally, the grinding force is considered a key indicator of the material removal process, which directly affects the machining efficiency, the quality of the machined surface, and the durability of the grinding wheel [9].

In a broader perspective, the values of the grinding force components obtained experimentally allow for the creation of mathematical models, and the force model is the simplest and most versatile mathematical expression describing the mechanical behaviour of material removal [9]. As shown by the comparative analysis presented in the study [9], mathematical models of the grinding force can come from various sources, including experimental data, empirical formulas, or theoretical analyses. It should be remembered that modelling the grinding force is a complex process, and the factors influencing the magnitude of the grinding force can include the physical properties of the machined material, the shape, size, and number of abrasive grains, grinding parameters, cooling and lubrication conditions, and the type of grinding process [10,11,12].

It is worth noting that grinding force models can be used in further research to model the dynamic grinding process. An example of such use is the model of the external plunge grinding process [13,14,15], the process of longitudinal cylindrical external grinding [16], and the process of peripheral surface grinding [17].

Therefore, this article presents research work, as a result of which two mathematical models were determined to predict the components of the grinding force during the sharpening of the rake surfaces of hob cutters. The research began with experimental studies, the conditions and methodology of which, as well as the results, are described in Section 2 and Section 3, respectively. The first of the two mathematical models presented in Section 4 is a modification of the model proposed by Werner [18]. It is worth noting that this model is often used, also with appropriate modifications, by other researchers [12,19,20]. The second mathematical model in the form of a multiple regression function was determined on the basis of algorithm of the “acceptance and rejection” method [21]. In the final part of the work, a comparison of the results obtained based on calculations using the proposed mathematical models with the results obtained experimentally was made. The work concludes with a summary and conclusions.

## 2. The Conditions and Methodology of the Research

**Figure 1:**
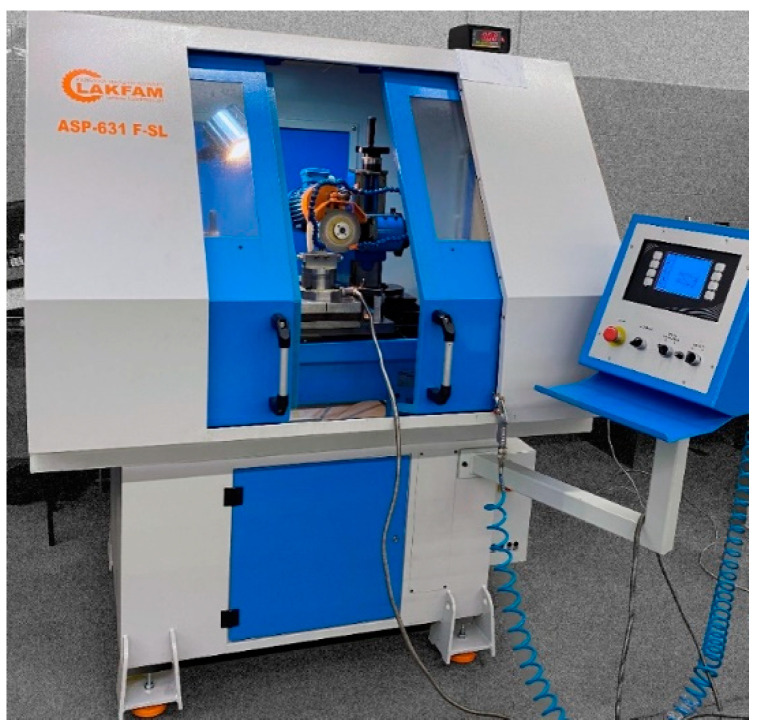
A general view of the ASP-631 F-SL tool grinder (Lakfam, Kowary, Poland).

The experimental studies were conducted on the ASP-631 F-SL tool grinder (Lakfam, Kowary, Poland), a general view of which is shown in Figure 1. The ASP-631 F-SL is a numerically controlled grinder in three axes for the automatic grinding of cutting tools along straight and helical lines. As such, it can be used for sharpening the rake surfaces of hob cutters.

**Figure 2:**
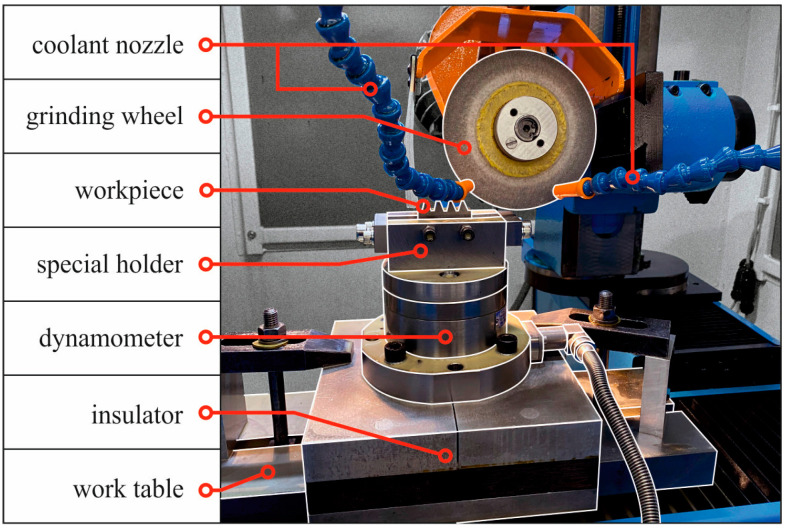
A view of the workspace of the ASP-631 F-SL tool grinder.

In Figure 2, the workspace of the ASP-631 F-SL tool grinder is shown. As can be observed, a 4-component piezoelectric dynamometer type 9272 (Kistler, Winterthur, Switzerland) is mounted on the grinder table, previously separated from the table by a layer of insulator. The dynamometer was used to measure the components of the grinding force—the normal component *F_n_* and the tangential component *F_t_*. The signal from the dynamometer was transmitted to a four-channel amplifier Kistler type 5019A, then to the data acquisition card type KUSB-3108 (Keithley Instruments, Cleveland, OH, USA), which was finally connected to a PC. The Keithley quick DAQ software (version 1.6) was used to record the measurement signal.

A special holder for mounting the sample was attached to the upper surface of the dynamometer. During the tests, a sample made of high-speed steel HS6-5-2 with a hardness of 62 ± 1 HRC was ground. The shape of the sample in the part subjected to grinding had the outline of a gear, with dimensions corresponding to the dimensions of the cutting edge gear of a hob cutter with a module *m* = 3 mm and a pressure angle α = 20°, in accordance with ISO 53 and ISO 54. The geometric dimensions of the gear were selected according to PN 4468, taking into account the accuracy of the hob cutter edge in class B.

As the cutting tool, a T3 type grinding wheel with dimensions (*D* × *T* × *H*) 125 × 9/2 × 32 mm was used. The parameter *D* denotes the outer diameter of the grinding wheel, the parameter *T* is its thickness, and the parameter *H* specifies the diameter of the central hole. It is worth noting that due to the conical shape of the grinding wheel’s active surface, the thickness *T* varies from 2 mm to 9 mm. The grinding wheels were made of alumina with a vitrified bond and marked with the symbol 38A60KVBE (Norton, Koło, Poland).

During the tests, a constant grinding speed *v_s_*, six workpiece speeds *v_w_*, and three cutting depths *a_e_* were used, allowing for 18 sets of cutting parameters. The machining conditions used during the tests are summarized in Table 1.

Each grinding test began with 10 spark-out passes. Then, a grinding test was carried out by removing the machining allowance (depth of cut) *a_e_* in one work cycle consisting of two passes: a working (grinding) pass and a return pass. The working pass was performed in the counter-direction, while the return pass was performed in the co-direction, without disengagement.

**Figure 3:**
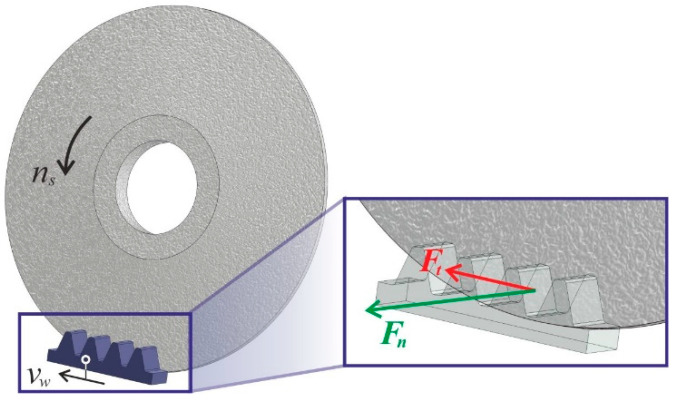
Distribution of grinding force components: *F_t_*—tangential component; *F_n_*—normal component; *n_s_*—grinding wheel rotational speed; *v_w_*—workpiece speed.

During the entire grinding test, the signal from the dynamometer was recorded using the previously discussed measurement setup. As mentioned earlier, two components of the grinding force were recorded—the normal component *F_n_* and the tangential component *F_t_* (Figure 3). The component *F_n_* is the force acting perpendicular to the ground surface, while the component *F_t_* is the force acting parallel to that surface.

As the cooling and lubricating fluid, a water–oil emulsion based on AGIP Aquamet 104 Plus oil in 5% concentration was used. Aquamet 104 Plus is a semi-synthetic emulsifying oil containing mineral and synthetic oils, as well as anticorrosive, detergent, and EP additives. The oil manufacturer states that its kinematic viscosity (at 40 °C) *ν* = 28 mm^2^/s, and its density (at 20 °C) *ρ* = 1.01 g/cm^3^. Emulsion was supplied to the grinding zone through two nozzles with a total flow rate of *Q* = 3 L/min.

## 3. Experimental Results

**Figure 4:**
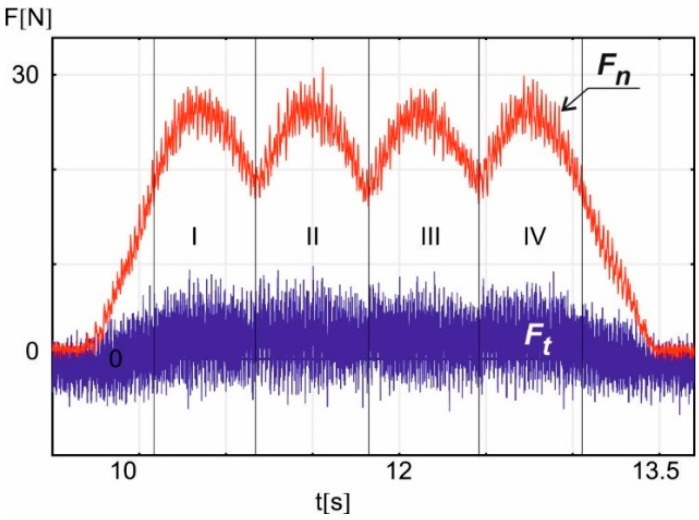
A sample time course of the tangential component *F_t_* and the normal component *F_n_* of the grinding force during the working pass; *a_e_* = 0.01 mm, *v_w_* = 0.9 m/min, sampling frequency 10 kHz.

In Figure 4, an example time plot of the tangential component *F_t_* and the normal component *F_n_* of the grinding force is presented, recorded during the working pass using the following machining parameters: *a_e_* = 0.01 mm and *v_w_* = 0.9 m/min.

As shown in the above graph, the shape of the course of both components of the grinding force depends on the length of contact between the grinding wheel and the workpiece. This length changes with the position of the grinding wheel relative to the gear-shaped sample with four teeth. For this reason, the graph is divided into four segments of force variability, corresponding to the four teeth of the hob cutter edge. These segments are numbered sequentially as I, II, III, and IV.

As can be observed in Figure 4, the highest value of the grinding normal force components in each of the four segments was obtained near the tooth tips, where the contact line is the longest. Similarly, the lowest value of the grinding force components was recorded at the tooth roots, where the contact line is the shortest.

It is worth noting that the presented force measurement is noisy. Fourier transformation showed that the noise frequency is close to the frequency associated with the variable dimensions of the gear-shaped sample.

The example time plot of the grinding force components shown in Figure 4 was obtained for one of the 54 measurements conducted under different machining conditions, considering three repetitions for each of the 18 sets of grinding parameters. To quantitatively compare the obtained results, the average value of *F_t_* and *F_n_* from segment II was calculated for each of the 54 working passes. This segment was taken from the full measurement, from the moment of reaching the minimum near segments I and II, to reaching the next minimum near segments II and III, as shown in Figure 4. Then, each of the three calculated average values for each of the 18 sets of grinding parameters was used to determine the average value of *F_t_* and *F_n_* representing a specific set of grinding parameters.

**Figure 5:**
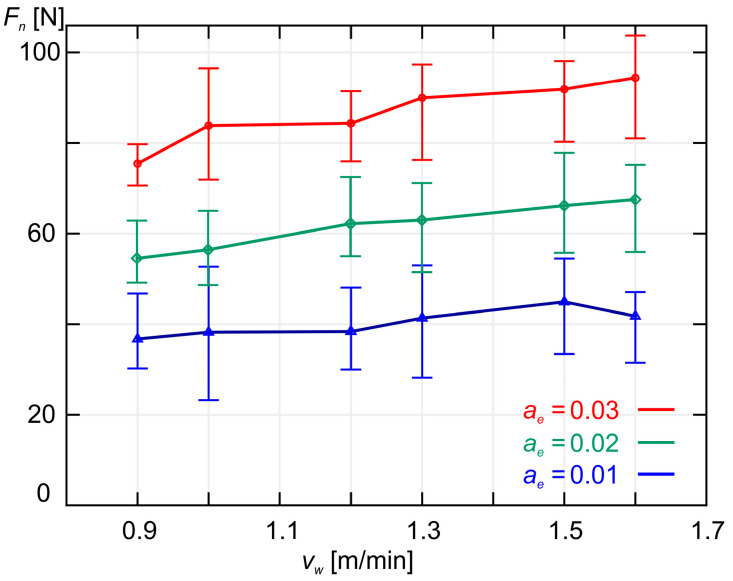
A plot of the average values of the normal component *F_n_* of the grinding force as a function of the workpiece speed *v_w_* for three different values of machining allowance *a_e_*.

In Figure 5, the average normal component *F_n_* of the grinding force is shown as a function of the workpiece speed *v_w_* and three different values of machining allowance *a_e_.* The smallest and largest values for each of the 18 sets of measurement results are also marked on the graph.

**Figure 6:**
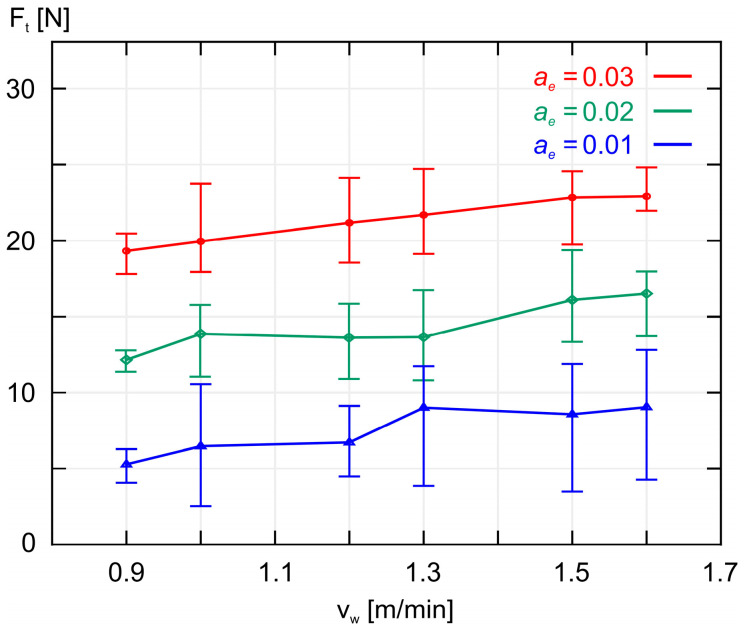
A plot of the average values of the tangential component *F_t_* of the grinding force as a function of the workpiece speed *v_w_* for three different values of machining allowance *a_e_*.

Similarly to the above, Figure 6 shows the average tangential component *F_t_* of the grinding force as a function of the workpiece speed *v_w_*.

**Figure 7:**
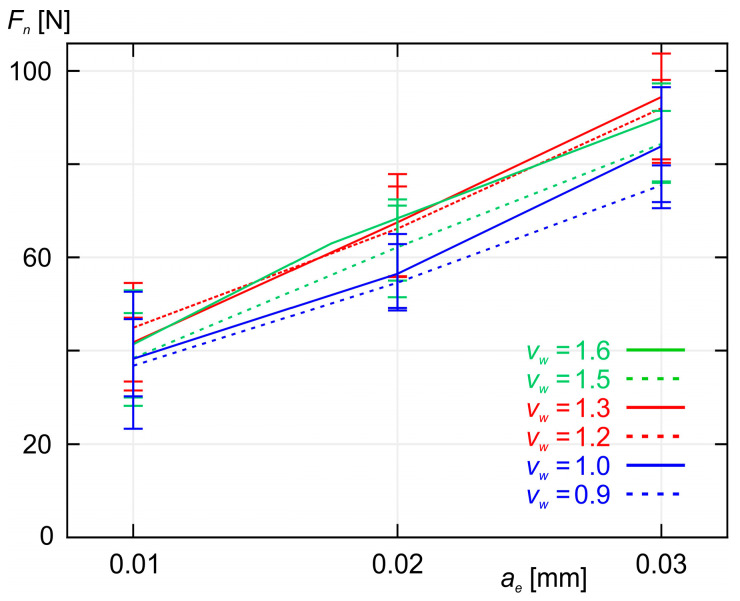
A plot of the average values of the normal component *F_n_* of the grinding force as a function of the machining allowance *a_e_* for six different values of workpiece speed *v_w_*.

**Figure 8:**
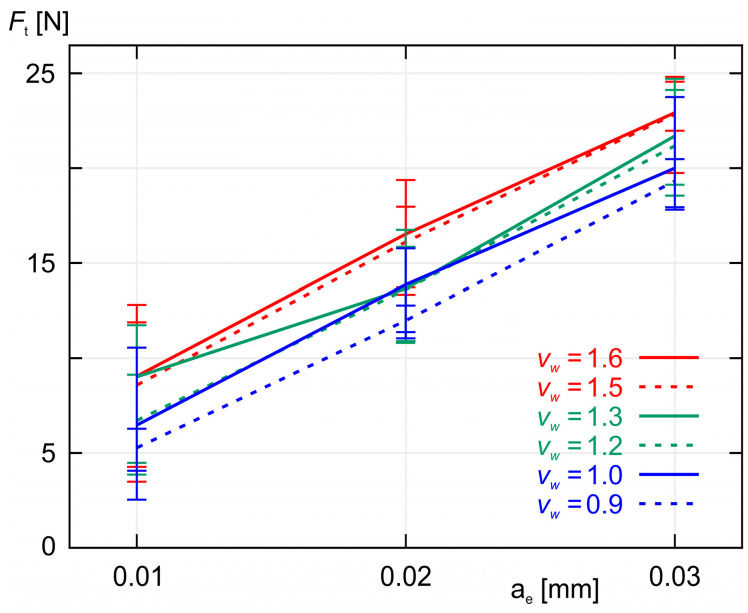
A plot of the average values of the tangential component *F_t_* of the grinding force as a function of the machining allowance *a_e_* for six different values of workpiece speed *v_w_*.

As shown in Figure 5 and Figure 6, increasing the workpiece speed *v_w_* at a constant grinding depth ae causes an increase in both the normal component *F_n_* and the tangential component *F_t_* of the grinding force. A similar effect is observed when increasing the grinding depth *a_e_* while maintaining a constant workpiece speed *v_w_*, as shown in Figure 7 and Figure 8. As expected, the smallest values of both *F_n_* and *F_t_* were achieved during grinding at the lowest workpiece speed (*v_w_* = 0.9 m/min), while simultaneously removing the smallest machining allowance (*a_e_* = 0.01 mm).

## 4. Force Models of Grinding Process

### 4.1. The Polynominal Model Force

The obtained experimental results allowed for the construction of a model for the normal component and the tangential component of the grinding force occurring during the hob cutter sharpening process. The models for the normal component *F_n_* available in the literature gave divergent results compared to the conducted experiment. Ultimately, the best-fitting model was accepted, whose general form as a polynomial for the normal component *F_n_* was presented by Werner in [18]:

(1) $$F_{n}=K\cdot {\left[C_{1}\right]}^{\gamma }\cdot {\left[\frac{Q_{w}^{\prime }}{v_{s}}\right]}^{2\in -1}\cdot {\left[a_{e}\right]}^{1-\in }\cdot {\left[d_{s}\right]}^{1-\in },$$

where *K* is the proportionality factor, *C*_1_ is the cutting edge density, *γ* is an exponent depending on the grinding parameter, *Q*^’^*_w_* is the specific material removal rate, *v_s_* is the grinding wheel peripheral speed, ∈ is an exponent depending on the workpiece material, *a_e_* is a grinding depth, and *d_s_* is the diameter of the grinding wheel.

Based on Equation (1), our own mathematical model was proposed, encompassing both components of the grinding force, in the form of the equation:

(2) $$F_{n,t}\left(v_{w},a_{e}\right)=K\cdot {\left(\frac{v_{w}}{v_{s}}\right)}^{2f-1}\cdot {\left(\frac{a_{e}}{D}\right)}^{f}\cdot D,$$

where

*K*—grinding coefficient, N/mm;
*f*—exponential coefficient;
*v_w_*—workpiece speed, m/min;
*v_s_*—grinding wheel peripheral speed, m/s;
*a_e_*—grinding depth, mm;
*D*—grinding wheel outer diameter, mm.

Based on the results obtained from the experimental studies, the parameters of the mathematical model were calculated using the least squares method, in the Mathematica software (version 8). After substituting them into Equation (2), two equations for calculating the components of the grinding force were obtained:

(3) $$F_{n}\left(v_{w},a_{e}\right)=506.751\cdot {\left(\frac{v_{w}}{v_{s}}\right)}^{0.3622}\cdot {\left(\frac{a_{e}}{D}\right)}^{0.6811}\cdot D,$$

(4) $$F_{t}\left(v_{w},a_{e}\right)=598.263\cdot {\left(\frac{v_{w}}{v_{s}}\right)}^{0.5995}\cdot {\left(\frac{a_{e}}{D}\right)}^{0.7998}\cdot D,$$

where

*v_s_* = 30.1 m/s;
*D* = 125 mm.

The multiple regression coefficient for these procedures was estimated at 0.9989 for normal force *F_n_* and 0.9946 for tangential force *F_t_*.

### 4.2. The Regression Analysis Procedure

To determine the mathematical relationships for calculating the normal component *F_n_* and the tangential component *F_t_* of the grinding force, the SKZ program (version 1.0) was used, which has been successfully developed and utilized by the authors in other research works [22,23,24]. The measured values from the experimental studies were used as input data.

The SKZ program determines the coefficients of the multiple regression equation using a regression analysis procedure based on the “acceptance and rejection” method [21]. The selection of critical values of the statistic (*F_kr_*) is made at a significance level of α = 0.4, and after determining the regression function, it is changed to a value of α = 0.1. Calculations begin with the simplest regression function, sequentially adding new terms. When a newly added term reduces the significance of an already introduced term, it is considered insignificant and removed from the regression function. After introducing all significant terms, a panel with a preview of the calculation results is displayed on the monitor screen.

Ultimately, the function of the research object took the following general form:

(5) $$F_{n,t}=F_{0}\cdot {\left(v_{w}\right)}^{f_{v}}\cdot {\left(a_{e}\right)}^{f_{a}},$$

where

*F*_0_—constant of the equation;
*f_v_*, *f_a_*—exponents of the equation.

The constants and coefficients determined from the calculations allowed for obtaining the relationship for the normal component *F_n_* of the grinding force with a multiple correlation coefficient *R* = 0.8893:

(6) $$F_{n}=876.6\cdot {\left(v_{w}\right)}^{0.338}\cdot {\left(a_{e}\right)}^{0.685}.$$

Similar calculations allowed for obtaining the relationship for the tangential component *F_t_* of the grinding force with a multiple correlation coefficient *R* = 0.8604:

(7) $$F_{t}=775.9\cdot {\left(v_{w}\right)}^{0.529}\cdot {\left(a_{e}\right)}^{1.052}.$$

The significance of the obtained multiple correlation coefficients *R* was determined using the Fisher–Snedecor F-test. For this purpose, the test values of the *F* factor were calculated and compared with the critical values *F_kr_*. Since the *F* values are greater than the *F_kr_* values, the correlation coefficients should be considered significant, which, as indicated in [25], entails the agreement (at a significance level of α = 0.1) of the regression function equation with the results of the conducted experimental studies.

### 4.3. Comparison of Models and Experiment Results

Despite the satisfactory values of the multiple correlation coefficients for the developed mathematical models, they were also verified by comparing the calculated values of the normal component *F_n_* and the tangential component *F_t_* of the grinding force with the values obtained during the experimental studies. For this purpose, the values of the grinding force components *F_n_* and *F_t_* were calculated for the 18 sets of grinding parameters used in the studies using Equations (6) and (7). Additionally, the values of both force components were also determined based on Equations (3) and (4) of the polynomial model.

**Figure 9:**
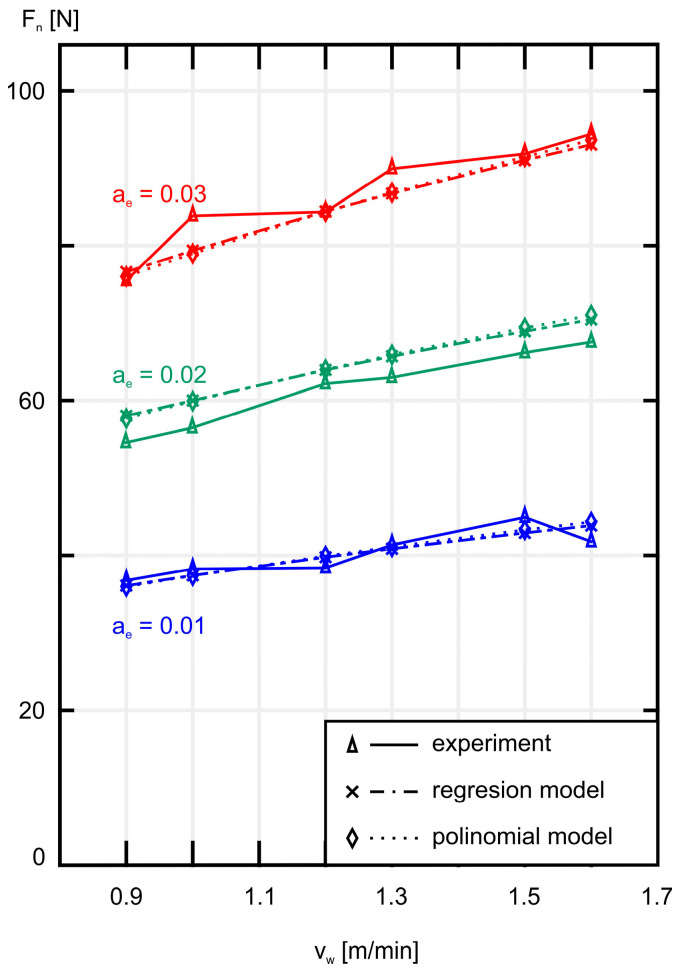
A plot of the experimental (solid line), regression model (point line), and polynomial model (dotted line) values of mean normal forces *F_n_* versus workpiece speed *v_w_* for three grinding depths *a_e_*.

**Figure 10:**
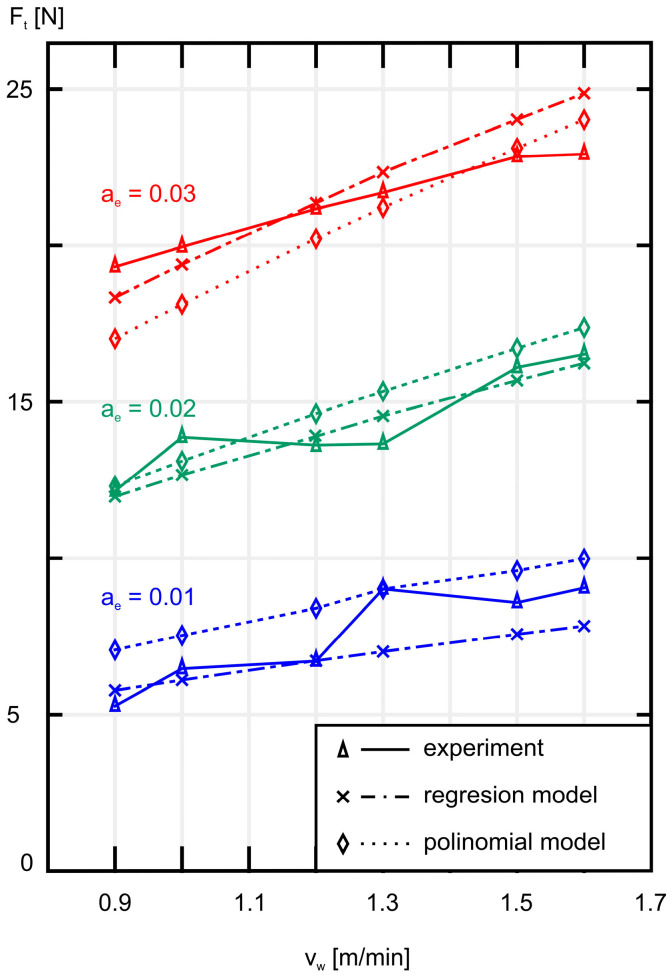
A plot of the experimental (solid line), regression model (point line), and polynomial model (dotted line) values of mean tangential forces *F_t_* versus workpiece speed *v_w_* for three grinding depths *a_e_*.

In Figure 9, a comparison of the experimental results with the results obtained from the mathematical models describing the normal component *F_n_* of the grinding force is presented. A similar graph showing the tangential component *F_t_* of the grinding force is shown in Figure 10.

The calculation results of *F_n_* and *F_t_* obtained from the two presented models are summarized in Table 2 and Table 3, respectively. In the case of the normal component *F_n_* of the grinding force, the relative errors of the models are larger compared to those obtained for the tangential component *F_t_* of the grinding force. The largest relative error for *F_n_* in the regression model occurs at a grinding depth *a_e_* = 0.01 mm (($\bar{\left|∆\right|}$) = 3.820%), while for the polynomial model it occurs at a grinding depth *a_e_* = 0.02 mm (($\bar{\left|∆\right|}$) = 3.922%). Nevertheless, the overall average errors for both models remain relatively small, amounting to 3.241% for the regression model and 3.228% for the polynomial model.

It is worth noting that the largest relative error for the models of the tangential component *F_t_* of the grinding force is observed in the regression model (($\bar{\left|∆\right|}$) = 2.016%) and the polynomial model (($\bar{\left|∆\right|}$) = 2.755%) at the same grinding depth *a_e_* = 0.01 mm, which is the smallest. The overall average error for both models is quite small, amounting to 1.409% for the regression model and 1.727% for the polynomial model.

Analyzing Table 2 and Table 3, as well as Figure 9 and Figure 10, it can be seen that both models effectively replicate the experimental results. In the case of the normal component *F_n_*, the results obtained from both models are almost identical, while for the tangential component *F_t_*, the results show a very small relative error despite the irregular course of the experimental results. Therefore, the results calculated based on the presented models and the results obtained from the experiment are consistent with each other.

## 5. Conclusions

The article presents mathematical models in the form of multiple regression functions and polynomial functions, enabling the calculation of the normal component *F_n_* and the tangential component *F_t_* of the grinding force during the sharpening of the rake surfaces of hob cutters. The models were determined based on the results of experimental studies.

Based on the obtained research results, it can be concluded that:

1. Both the experimental results and the results obtained from the calculations based on the models indicate that as the grinding depth *a_e_* increases, the values of the grinding force components (*F_n_* and *F_t_*) also increase, which was expected. A similar relationship was found when the workpiece speed *v_w_* was increased.
2. In the case of the normal component *F_n_*, the overall percentage average errors for both models remain small, amounting to 3.241% for the regression model and 3.228% for the polynomial model. For the tangential component *F_t_*, the overall percentage average error for both models is small, amounting to 1.409% for the regression model and 1.727% for the polynomial model.
3. The values of both grinding force components calculated based on the mathematical models are consistent with the results obtained from the experiment. In the case of the *F_n_* component, the calculated values in both cases deviate insignificantly from the experimental values, while for the *F_t_* component, the results show a very small relative error despite the irregular course of the experimental results, which should be considered very satisfactory. Additionally, the high value of the multiple regression coefficient for both models demonstrates compliance with the experimental results.
4. The developed models provide a good basis for analyzing the hob cutter sharpening process with variable grinding parameters, and can also be used in modelling the dynamic grinding process.

## Figures and Tables

**Table 1:** Grinding conditions used during experimental tests.

Workpiece material	HS6-5-2, carburized and hardened with 62 ± 1 HRC
Grinding wheel	38A60KVBE
Grinding wheel rotational speed	*n_s_* = 4600 rpm
Grinding wheel peripheral speed	*v_s_* = 30.1 m/s
Workpiece speed	*v_w_*_1_ = 0.9 m/min *v_w_*_2_ = 1.0 m/min *v_w_*_3_ = 1.2 m/min *v_w_*_4_ = 1.3 m/min *v_w_*_5_ = 1.5 m/min *v_w_*_6_ = 1.6 m/min
Machining allowance (working engagement, grinding depth)	*a_e_*_1_ = 0.01 mm *a_e_*_2_ = 0.02 mm *a_e_*_3_ = 0.03 mm
Number of passes: grinding/return	1/1
Grinding direction	Up grinding
Dresser	Single-point diamond dresser
Dresser weight	*Q_d_* = 1.0 kt (0.2 g)
Grinding wheel peripheral speed while dressing	*v_sd_* = 30.1 m/s
Dressing allowance	*a_d_* = 0.01 mm
Axial table feed speed while dressing	*v_fd_* = 40 mm/min
Number of dressing passes	*i_d_* = 10
Environments	WET—conventional flood method
Coolant	AGIP Aquamet 104 Plus in a 5% concentration
Coolant flow rate	*Q* = 3 L/min

**Table 2:** The values of the normal component *F_n_* of the grinding force obtained experimentally and calculated based on models.

Input Data			Regression Model					Polynomial Model				
*v_w_* [m/min]	*a_e_* [mm]	Normal Force *F_n_* [N] (Experiment)	Normal Force *F_n_* [N] (Regression Model)	Error |Δ|	Percentage Error $\frac{\left|\Delta \right|}{F_{t}}$ · 100%	Average Error $\sum \frac{\left|\Delta_{i}\right|}{n}$	Percentage Average Error $\sum \frac{\left|\Delta_{i}\right|}{F_{t}n}*100\%$	Normal Force *F_n_* [N] (Polynomial Model)	Error |Δ|	Percentage Error $\frac{\left|\Delta \right|}{F_{t}}$ · 100%	Average Error $\sum \frac{\left|\Delta_{i}\right|}{n}$	Percentage Average Error $\sum \frac{\left|\Delta_{i}\right|}{F_{t}n}*100\%$
0.9	0.01	37.799	36.086	1.713	4.533	1.597	3.820	35.968	1.831	4.844	1.620	3.896
1.0		39.294	37.394	1.900	4.835			37.367	1.927	4.903		
1.2		39.438	39.771	0.333	0.844			39.918	0.480	1.218		
1.3		42.405	40.862	1.543	3.640			41.092	1.313	3.095		
1.5		45.966	42.886	3.080	6.700			43.278	2.688	5.847		
1.6		42.817	43.832	1.015	2.371			44.302	1.485	3.468		
0.9	0.02	55.135	58.015	2.880	5.224	2.306	3.770	57.670	2.535	4.598	2.424	3.922
1.0		57.044	60.118	3.074	5.389			59.913	2.869	5.030		
1.2		62.764	63.940	1.176	1.873			64.003	1.239	1.975		
1.3		63.557	65.693	2.136	3.361			65.886	2.329	3.664		
1.5		66.745	68.949	2.204	3.302			69.391	2.646	3.964		
1.6		68.105	70.469	2.364	3.471			71.032	2.927	4.298		
0.9	0.03	75.480	76.588	1.108	1.469	1.841	2.132	76.013	0.533	0.706	1.611	1.868
1.0		83.828	79.365	4.463	5.324			78.969	4.859	5.796		
1.2		84.340	84.410	0.070	0.083			84.360	0.020	0.024		
1.3		89.932	86.725	3.207	3.566			86.842	3.090	3.436		
1.5		91.842	91.022	0.820	0.892			91.462	0.380	0.414		
1.6		94.408	93.030	1.378	1.460			93.625	0.783	0.830		
Total average error:						1.915	3.241	Total average error:			1.885	3.228

**Table 3:** The values of the tangential component *F_t_* of the grinding force obtained experimentally and calculated based on models.

Input Data			Regression Model					Polynomial Model				
*v_w_* [m/min]	*a_e_* [mm]	Tangential Force *F_t_* [N] (Experiment)	Tangential Force *F_t_* [N] (Regression Model)	Error |Δ|	Percentage Error $\frac{\left|\Delta \right|}{F_{t}}$ · 100%	Average Error $\sum \frac{\left|\Delta_{i}\right|}{n}$	Percentage Average Error $\sum \frac{\left|\Delta_{i}\right|}{F_{t}n}*100\%$	Tangential Force *F_t_* [N] (Polynomial Model)	Error |Δ|	Percentage Error $\frac{\left|\Delta \right|}{F_{t}}$ · 100%	Average Error $\sum \frac{\left|\Delta_{i}\right|}{n}$	Percentage Average Error $\sum \frac{\left|\Delta_{i}\right|}{F_{t}n}*100\%$
0.9	0.01	5.280	5.776	0.496	1.311	0.855	2.016	7.069	1.789	4.732	1.112	2.755
1.0		6.476	6.107	0.369	0.940			7.529	1.053	2.681		
1.2		6.719	6.725	0.006	0.015			8.399	1.680	4.260		
1.3		9.026	7.016	2.010	4.740			8.812	0.214	0.505		
1.5		8.587	7.568	1.019	2.218			9.601	1.014	2.207		
1.6		9.060	7.830	1.230	2.872			9.980	0.920	2.149		
0.9	0.02	12.501	11.975	0.526	0.954	0.724	1.185	12.305	0.196	0.356	0.628	1.025
1.0		14.272	12.662	1.610	2.823			13.107	1.165	2.042		
1.2		14.013	13.944	0.069	0.111			14.621	0.608	0.969		
1.3		14.053	14.547	0.494	0.777			15.340	1.287	2.025		
1.5		16.572	15.691	0.881	1.320			16.714	0.142	0.213		
1.6		17.001	16.236	0.765	1.124			17.373	0.372	0.547		
0.9	0.03	19.338	18.346	0.992	1.315	0.901	1.025	17.018	2.320	3.074	1.163	1.402
1.0		19.975	19.397	0.578	0.689			18.128	1.847	2.204		
1.2		21.197	21.361	0.164	0.195			20.221	0.976	1.157		
1.3		21.716	22.285	0.569	0.633			21.215	0.501	0.557		
1.5		22.865	24.038	1.173	1.277			23.116	0.251	0.273		
1.6		22.945	24.872	1.927	2.042			24.028	1.083	1.147		
Total average error:						0.827	1.409	Total average error:			0.968	1.727

## Data Availability

Data are contained within the article.
